# Preoperative radiographic and clinical factors associated with postoperative floating of the lesser toes after resection arthroplasty for rheumatoid forefoot deformity

**DOI:** 10.1186/s12891-019-2462-y

**Published:** 2019-02-19

**Authors:** Tomohiro Onodera, Hiroaki Nakano, Kentaro Homan, Eiji Kondo, Norimasa Iwasaki

**Affiliations:** 1 Department of Orthopaedic Surgery, Hokkaido University Graduate School of Medicine, Kita 15, Nishi 7, Kita-Ku, Sapporo, Hokkaido 060-8638 Japan; 2 Department of Advanced Therapeutic Research for Sports Medicine, Hokkaido University School of Medicine, Sapporo, Japan

**Keywords:** Rheumatoid arthritis, Forefoot deformity, Hallux valgus, Postoperative floating

## Abstract

**Background:**

This study aimed to clarify the characteristics associated with postoperative floating of the lesser toes, especially focusing on the medial and lateral lessor toes, after arthrodesis of the first metatarsophalangeal joint and resection arthroplasty of the lessor toes in rheumatoid forefoot deformity.

**Methods:**

Fourty-seven feet of 43 people who underwent resection arthroplasty of the metatarsal head of the lesser toes for rheumatoid arthritis of the metatarsophalangeal joints were included. We retrospectively evaluated the preoperative radiographic findings and clinical characteristics of the patients, and the occurrence of postoperative floating of the lesser toes. The mean duration of follow-up was 36.5 (range 12 to 114) months.

**Results:**

Preoperative dislocation grades of the second and third toes that demonstrated postoperative floating were significantly higher than those of toes that did not experience postoperative floating. The hallux valgus deformity before surgery was significantly more severe in toes with postoperative floating of the second and third lessor toes than those with no floating (*p* < 0.05). In addition, the Japanese Society for Surgery of the Foot (JSSF) hallux scale scores before surgery in toes with postoperative floating of the fourth and fifth lessor toes were significantly worse than those in non-dislocating toes (p < 0.05).

**Conclusions:**

The preoperative condition of the first metatarsophalangeal joint, including hallux valgus deformity, pain, range of motion, activity of daily living, and function is significantly different between postoperative floating of the lesser toes and non-floating of them after resection arthroplasty for rheumatoid forefoot deformity.

## Background

Rheumatoid arthritis frequently causes painful forefoot deformities that interfere with standing and walking [1, 2]. The prevalence of forefoot deformities in adults with chronic RA has been reported as approximately 90% [3, 4]. Therefore, management of patients with rheumatoid arthritis should include evaluation and care of the forefoot deformity. The metatarsophalangeal (MTP) joints are the most common sites affected by RA. Deformity or dislocation of MTP joints frequently result in decreasing the quality of life in RA patients [5]. Dislocation of MTP joints in the lesser toes are also known to disrupt joint function and lead to painful callosities. Although resection arthroplasty of the metatarsal heads of the lesser toes does not lead to improvement in gait or function and is often associated with impairment of push-off [6], resection arthroplasty of the metatarsal heads of the lesser toes by various operative techniques generally results in good long-term clinical outcomes [5, 7, 8].

Although joint-preserving procedures are commonly performed in patients with controllable RA, postoperative floating of the lesser toes, occurring as a complication following these surgeries, is reported to be a risk factor for unsatisfactory clinical outcomes [9]. Previous studies have shown that uneven and insufficient bone resection and preoperative MTP joint dislocation are risk factors for postoperative floating of the lesser toes after resection arthroplasty of the metatarsal heads of the lesser toes [10, 11]. However, the anatomic factors for postoperative floating of the lesser toes after resection arthroplasty of the metatarsal heads of the lesser toes are not fully understood.

Ouzounian TJ et al. clarified that second and third tarsometatarsal (TMT) joint is stable compared with lateral TMT joint (cuboid to fourth and fifth metatarsal bone) [12]. Based on the anatomical and functional characteristics, the foot is categorized into 2 parts, the medial and lateral columns. The medial column is composed of stable joints that maintain foot stability, while the lateral column consists of mobile joints that are responsible for the springing function of the foot. We speculated that each column is possibly affected by different pathologies in rheumatoid forefoot deformity.

Therefore, we hypothesized that the characteristics associated with postoperative floating of the lesser toes may be different between the medial and lateral lessor toes. The aim of this study was to clarify the characteristics for postoperative floating of the lesser toes, especially focusing on the differences between the medial and lateral lessor toes, after resection arthroplasty for rheumatoid forefoot deformity.

## Methods

### Subjects

Institutional review board approval of Hokkaido University Hospital was obtained prior to initiation of this study. Between 2007 and 2016, a total of 43 patients (47 ft) at a single center who were followed up for at least 12 months, and were diagnosed with rheumatoid forefoot deformities by the senior authors of this paper, that required arthrodesis of the first MTP joint, resection arthroplasty of the metatarsal heads of the lesser toes, and surgical repair of hammer toe deformity, were included in this study. One of the authors reviewed the medical records at our institution to identify patients with RA who underwent resection arthroplasty of the metatarsal heads of the lesser toes. Eligible patients were identified based on their diagnosis (RA) and the surgical procedure performed (resection arthroplasty of the metatarsal head of the lesser toes). All surgeries were performed by four senior surgeons. The indications for surgery in all patients were metatarsalgia associated with hallux valgus or joint destruction caused by RA. The patients who had no first MTP joint destruction and the hallux valgus deformity was less than 50 degree were performed joint-preserving surgery and were excluded in this study. The mean age of the subjects at the time of surgery was 61.8 (39–80) years. All the patients were women, and the follow-up period was 36.5 (12–114) months. All surgeries were performed by the same senior surgeon.

### Surgical procedure

**Fig. 1:**
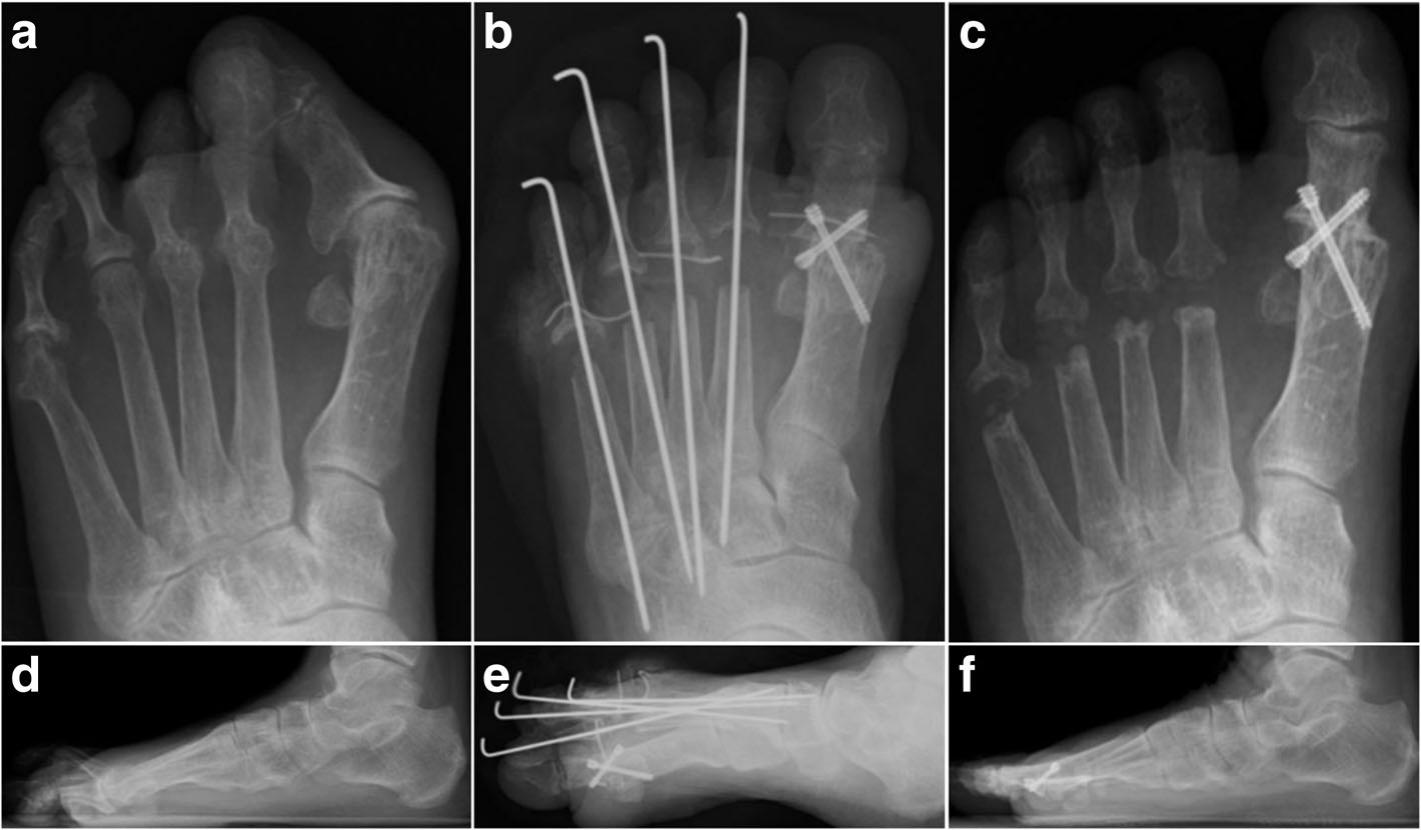
Representative radiographs. Preoperative AP (**a**) and lateral (**d**) radiographs showing typical forefoot deformities including hallux valgus, claw toe and MTP subluxation of second to fourth toes, and the quintus varus. Postoperative AP (**b**) and lateral (**e**) radiographs showing acceptable alignment of first MTP joint and no MTP subluxation of lessor toes. Radiographs five years after operation still indicate acceptable alignment (**c**, **f**)

The operative methods were precisely described in our previous study [13, 14]. Arthrodesis was performed through a dorsal longitudinal incision centred over the first MTP joint. The inclination of the first metatarsal in preoperative X-ray was referred to achieve an optimal angle of dorsiflexion of the first MTP joint [14]. The first MTP joint was fused using cannulated cancerous screw or Kirschner wires (K-wires) (Fig. 1). An arthrodesis of the interphalangeal (IP) joints of the second to fourth toes or second to fifth toes was performed. The metatarsal heads of the lesser rays were resected at the anatomic neck of the involved metatarsal via Lipscomb’s dorsal approaches [15].

The patients were allowed full weight-bearing at 2 weeks postoperatively, using a special shoe that allowed them to bear weight on their hindfoot. The special shoe was worn until fusion of the arthrodesis was confirmed.

### Radiographic evaluation and clinical evaluation

X-rays were taken during full weight-bearing, using lateral and standard dorsoplantar views before surgery and at the final follow up. Preoperative and postoperative evaluation was performed by one author. The valgus angle between the first metatarsal bone and the proximal phalanx (HVA: hallux valgus angle), the intermetatarsal angle between the first and second metatarsals (M1-2A: first-second intermetatarsal angle), and the intermetatarsal angle between the first and fifth metatarsals (M1-5A: first-fifth intermetatarsal angle), and the shortening distance of the metatarsal bone were measured on AP radiographs before surgery. The HVA were measured on AP radiographs after surgery. Tibial sesamoid position is measured on weight-bearing AP radiographs on a scale from 1 to 7 as described by Hardy and Clapham before and after surgery [16]. The joint destruction of the first MTP joint was evaluated by using the Larsen grading system [17]. The Larsen Grading Scale is used to determine the extent of radiographic changes due to rheumatoid arthritis. The scale score from 0 to 5 are based on the amount of spacing between joints, bony outlines, and level of joint erosion on radiographs. Total arc of first MTP joint was measured before surgery using a standard mechanical goniometer. Dislocation of MTP joints were graded from 0 (no dislocation) to 3 (complete dislocation) using preoperative weight-bearing lateral radiographs and the findings of inspection [18]. Since preoperative grading system of the dislocation of the MTP joints is based on the metatarsal head, it is impossible to grade if it is resected (especially grade 2 and 3). Therefore non-grounded toes while weight-bearing were defined as postoperative floating toes. All toes were divided into non-floating or postoperative floating groups. All the participating feet were also evaluated after categorizing them based on whether the medial, second and third toes, or lateral, fourth and fifth toes, lessor toes were affected. Regarding the foot with floating toes in both columns, we compared both columns by radiological and physical findings and divided into each group. Feet with absence of postoperative floating of both toes were classified as the non-floating group, while those with postoperative floating of one or both of the toes were defined as the postoperative floating group.

Before analysis of the X-ray films, intraobserver reproducibility was calculated by one author based on 5 consecutive measurements using the same X-ray film. Intraobserver consistency in all measurements was assessed by calculating the coefficient of variation (%, CV), which equals the standard deviation divided by the mean value. CVs were calculated for all the measurements. The CV of all measurements from single observer ranged from 1.5 to 3.1%. All of the variations were less than 10%, which was considered acceptable for comparison study purposes. To reduce data variation, a single investigator performed all study measurements.

Postoperative clinical outcomes were evaluated on the basis of data obtained during interviews and physical examinations. The Japanese Society for Surgery of the Foot (JSSF) hallux metatarsophalangeal-interphalangeal scale including hallux valgus deformity, pain, range of motion, activity of daily living, and function were used to assess clinical outcomes for the entire foot [19].

### Statistical analysis

Differences between non-floating and postoperative-floating were analysed by applying the unpaired *t*-test or the Mann-Whitney *U* test for continuous variables and the chi-square test or Fisher’s exact probability test for categorical variables. Statistical analyses were performed using JMP Pro version 10.0 statistical software (SAS Institute, Cary, NC). Statistical differences were considered significant for values of *p* < 0.05.

## Results

Patient demographics divided by final status of their toes are shown in Table 1. There was no significant difference between two groups in BMI (body mass index), follow-up period, duration of disease, CRP (C-reactive protein), MMP-3 (matrix metalloproteinase 3), DAS28-CRP (disease activity score in 28 joints), methotrexate or prednisolone user, and preoperative total arc of first MTP joint. Although non-floating patients showed the tendency of higher rate of bDMARDs (biological disease-modifying antirheumatic drug) use (*P* = 0.06), there were no significant difference between non-floating and postoperative floating patients.

**Table 1:** Patient demographics. Values are mean (SD) unless otherwise indicated

	Non-floating	Postoperative floating	*p* value
Age (years)	61.4 (10.1)	62.2 (6.5)	0.76
Sex			
Male (%)	0 (0%)	0 (0%)	
Female (%)	22 (100%)	25 (100%)	
BMI	21.3 (4.1)	20.5 (3.2)	0.45
Clinical follow-up (months)	36.0 (29.0)	36.9 (28.0)	0.92
Duration of disease (years)	17.8 (8.9)	22.6 (10.7)	0.12
CRP (mg/dl)	0.52 (1.1)	0.26 (0.2)	0.40
MMP-3 (ng/ml)	130.7 (88.9)	100.4 (82.2)	0.42
DAS28-CRP	3.40 (1.6)	3.27 (1.5)	0.89
bDMARD use (%)	11 (50%)	6 (24%)	0.06
MTX user (%)	15 (68%)	15 (60%)	0.56
PSL user (%)	19 (86%)	15 (60%)	0.13
Total arc of first MTP joint(°)	23.9 (19.2)	26.3 (16.2)	0.35

*BMI* body mass index, *CRP* C-reactive protein, *MMP-3* matrix metalloproteinase 3, *DAS28-CRP* disease activity score in 28 joints, *bDMARD* biological disease-modifying antirheumatic drug, *MTX* methotrexate, *PSL* prednisolone, *MTP* metatarsophalangeal

There was no delayed union in the first MTP joints of any of the patients. No cases of infection or delayed wound healing were observed in either group. The number of postoperative floatings and non-floatings in each toe are listed in Table 2.

**Table 2:** Number and proportion of postoperative floating and non-floating cases for each toe. Data are presented as number (%)

	Non-floating	Postoperative floating
Number of cases		
2nd toe	26 (60%)	17
3rd toe	25 (58%)	18
4th toe	23 (53%)	20
5th toe	19 (44%)	24

The preoperative dislocation grades of each lesser toe are shown in Table 3. The preoperative dislocation grade in postoperatively dislocated second and third toes were significantly worse than those of non-floating toes. The resection lengths of the metatarsal head in each toe are also shown in Table 3. There were no significant differences in resection lengths between any of the floating and non-floating lesser toes.

**Table 3:** Parameters of postoperative floating and non-floating cases for each toe. Values are mean (SD) unless otherwise indicated

	Non-floating		Postoperative floating		*p* value
	Grade 0–2	grade 3	Grade 0–2	Grade 3	
Number of cases					
2nd toe (%)	15 (35%)	11 (26%)	1 (2%)	16 (37%)	0.01
3rd toe (%)	12 (28%)	13 (30%)	1 (2%)	17 (40%)	0.02
4th toe (%)	13 (30%)	10 (23%)	4 (9%)	16 (37%)	0.08
5th toe (%)	14 (33%)	5 (12%)	15 (35%)	9 (21%)	0.40
Resection length (mm)					
2nd toe	18.0 (7.3)		19.9 (7.7)		0.47
3rd toe	19.6 (6.3)		19.8 (8.5)		0.93
4th toe	19.5 (5.6)		18.8 (7.2)		0.75
5th toe	16.6 (6.2)		14.8 (6.7)		0.42

Regarding postoperative floating in the medial lessor toes, the preoperative and postoperative hallux valgus deformity in postoperative floating toes was significantly more severe as compared to that in non-floating toes (52.8 vs. 38.4, *p* = 0.01; 14.1 vs. 8.0, *p* = 0.02) (Table 4). In the lateral lessor toes, preoperative JSSF hallux scale scores in the postoperative floating group was significantly lower than that in the non-floating group (31.7 vs. 44.2, *p* < 0.04) (Table 5).

**Table 4:** Parameters for postoperative floating of the medial column. Values are mean (SD) unless otherwise indicated

	Non-floating (*n* = 30)	Postoperative floating (*n* = 17)	*p* value
Radiographic (°)			
Pre-operative Hallux valgus	38.4 (17.7)	52.8 (13.0)	0.01
Pre-operative First-second intermetatarsal	12.2 (4.4)	16.9 (14.0)	0.28
Pre-operative First-fifth intermetatarsal	31.1 (8.4)	33.4 6.2)	0.35
Post-operative Hallux valgus	8.0 (7.6)	14.1 (7.8)	0.02
Pre-operative Hardy classification	6.2 (1.7)	5.9 (1.8)	0.71
Post-operative Hardy classification	4.4 (1.6)	3.8 (1.6)	0.22
Larsen grade	4.3 (1.1)	4.5 (1.1)	0.50
Clinical			
JSSF hallux scale	40.7 (14.0)	30.7 (16.0)	0.12
JSSF lesser scale	34.8 (11.7)	26.5 (10.1)	0.16

**Table 5:** Parameters for postoperative floating of the lateral column. Values are mean (SD) unless otherwise indicated

	Non-floating (*n* = 24)	Postoperative floating (*n* = 23)	*p* value
Radiographic (°)			
Hallux valgus	40.1 (16.7)	47.9 (18.1)	0.19
First-second intermetatarsal	12.3 (3.7)	15.6 (4.5)	0.31
First-fifth intermetatarsal	33.1 (5.9)	31.4 (8.1)	0.49
Post-operative Hallux valgus	8.1 (8.2)	12.6 (7.9)	0.11
Pre-operative Hardy classification	5.8 (1.9)	6.3 (1.6)	0.45
Post-operative Hardy classification	4.4 (1.9)	4.0 (1.4)	0.61
Larsen grade	4.3 (1.1)	4.5 (1.0)	0.45
Clinical			
JSSF hallux scale	44.2 (14.6)	31.7 (13.8)	0.04
JSSF lesser scale	34.1 (14.1)	30.3 (9.6)	0.50

## Discussion

The present study revealed that the hallux valgus deformity in postoperative floating of MTP joints in the medial lessor toes was significantly worse comparing with that in non-floating patients, whereas the clinical score of the first MTP joint is significantly poorer for postoperative floating of the lateral lessor toes comparing with that in non-floating patients. These results suggest that the poor condition of the first MTP joint is a characteristic for postoperative floating of lesser MTP joints in both sides.

Recent progress in the pharmacologic treatment for rheumatoid arthritis allows surgeons to perform MTP joint-preserving surgery. Several studies presented satisfactory outcomes with MTP joint-preserving surgery. However, its surgical indication depends on disease activity of RA, forefoot deformity and range of motion [20–22]. Resection arthroplasty is still needed for the patients who have severe deformities or disrupted joint. Although several recent studies indicated that the recurrence of hammer toe, shortening of the resection arthroplasty space, and the postoperative alignment of first ray were associated with worse clinical outcomes [10, 14, 23], the features with patient dissatisfaction after resection arthroplasty are still fully understood.

Regarding the relationship between postoperative satisfaction with the procedure and bone resection of the metatarsal bone, previous studies have shown that unsatisfactory postoperative results were associated with recurrence of hammer toe deformity [10], painful callosities [24], and uneven and insufficient bone resection [11]. Our results showed that bone resection length did not have a significant correlation with postoperative floating toes, whereas preoperative MTP joint dislocation grade was significantly worse in the postoperative floating group. Our results suggest that additional bone resection according to soft tissue condition or plantar soft tissue release is required to avoid postoperative floating toes in patients with severe preoperative dislocation of lesser MTP joints.

The severity of preoperative hallux valgus deformity and postoperative valgus alignment of the fused first MTP joint were significantly worse in the postoperative MTP floating in the medial column group as compared with the non-postoperative floating toes group. We speculated that the great toe bends laterally and physically pushes the medial lessor toes, resulting in severe preoperative MTP dislocations. Another suggested hypothesis is that past RA activity simply correlates with the severity of hallux valgus and preoperative MTP dislocation of the medial lessor toes. Hence, preoperative severity of hallux valgus deformity possibly affects the incidence of postoperative floating of MTP joints in the medial lessor toes.

Regarding postoperative floating toes in the lateral lessor toes, JSSF hallux scale scores were significantly worse in the postoperative floating group, whereas lesser JSSF scale scores were not significantly different. Our results also revealed that there was no significant difference between non-floating and postoperative floating group in the range of motion of first MTP joint before surgery. The JSSF hallux scale is based on deformity, activities of daily life, and the presence of callosities, pain and range of motion [19]. Recent studies suggested that forefoot kinematics are altered by dysfunctions associated with hallux valgus [25]. Load transfer over the metatarsal heads of the lesser toes is increased during the stance phase of gait [26–28]. It seems reasonable to suppose that the symptoms of the hallux condition, such as pain, callosity, restricted range of motion and dysfunction possibly lead to shifting their center of pressure laterally [29], resulting in excess dorsiflexion stress on the lateral lesser toes before surgery. we speculated that the preoperative gait which is the external load has exacerbated the degree of dislocation of the lateral MTP joints, which in turn causes postoperative-floating.

Several limitations must be considered when interpreting the present findings. The first is the small sample size, which is the result of the decreasing prevalence of severe forefoot deformity due to the frequent use of highly effective drugs, such as methotrexate and biologic agents, in patients with RA. A second limitation is the lack of evaluation of non-surgical patients. Evaluation of non-surgical treatment patients may be helpful for more in depth understanding of the pathology of rheumatoid forefoot deformity. Third limitation is that we didn’t evaluate postoperative DAS-CRP. Although the evaluation of preoperative DAS-CRP reflects disease activity, ideally it would be desirable to consider the evaluation of postoperative DAS-CRP. The fourth limitation is that the evaluation methods of the dislocation/floating of the toes are different before and after surgery. Preoperative grading system is simple and easy to understand. However, it is based on the location of proximal pharyngeal bone standardized by the metatarsal head. Therefore, we could not apply it for postoperative evaluations.

## Conclusions

The preoperative condition of the first metatarsophalangeal joint, including the severe hallux valgus deformity, poor clinical scoring possibly affect the incidence of postoperative floating of lessor toes after resection arthroplasty for rheumatoid forefoot deformity. Surgeons must consider that severe hallux valgus deformity may induce postoperative floating of the medial lesser toes and poor clinical score of the first metatarsophalangeal joint may induce postoperative floating of the lateral lesser toes.

## Abbreviations

bDMARD: Biological disease-modifying antirheumatic drug
BMI: Body mass index
CRP: C-reactive protein
CV: Coefficient of variation
DAS28-CRP: disease activity score in 28 joints
HVA: Hallux valgus angle
IP: Interphalangeal
JSSF: Japanese Society for Surgery of the Foot
MMP-3: Matrix metalloproteinase 3
MTP: Metatarsophalangeal
MTX: Methotrexate
PSL: Prednisolone
RA: Rheumatoid arthritis
